# Combined exposure to heavy physical workload and low job control and the risk of disability pension: A cohort study of employed men and women in Sweden

**DOI:** 10.1007/s00420-023-01983-8

**Published:** 2023-05-29

**Authors:** Kathryn Badarin, Tomas Hemmingsson, Melody Almroth, Daniel Falkstedt, Lena Hillert, Katarina Kjellberg

**Affiliations:** 1 Unit of Occupational Medicine, Institute of Environmental Medicine, Karolinska Institutet, Stockholm, Sweden; 2 Department of Public Health Sciences, Stockholm University, Stockholm, Sweden; 3 Centre for Occupational and Environmental Medicine, Region Stockholm, Stockholm, Sweden

**Keywords:** Ageing employee, Disability benefit, Heavy manual job, Heavy work, Decision authority, Musculoskeletal, Early exit, Physical health, Work ability, Work conditions

## Abstract

**Objective:**

To investigate the separate and combined effects of overall heavy physical workload (PWL) and low decision authority on all-cause disability pension (DP) or musculoskeletal DP.

**Methods:**

This study uses a sample of 1,804,242 Swedish workers aged 44–63 at the 2009 baseline. Job Exposure Matrices (JEMs) estimated exposure to PWL and decision authority. Mean JEM values were linked to occupational codes, then split into tertiles and combined. DP cases were taken from register data from 2010 to 2019. Cox regression models estimated sex-specific Hazard Ratios (HR) with 95% confidence intervals (95% CI). The Synergy Index (SI) estimated interaction effects.

**Results:**

Heavy physical workload and low decision authority were associated with an increased risk of DP. Workers with combined exposure to heavy PWL and low decision authority often had greater risks of all-cause DP or musculoskeletal DP than when adding the effects of the single exposures. The results for the SI were above 1 for all-cause DP (men: SI 1.35 95%CI 1.18–1.55, women: SI 1.19 95%CI 1.05–1.35) and musculoskeletal disorder DP (men: SI 1.35 95%CI 1.08–1.69, women: 1.13 95%CI 0.85–1.49). After adjustment, the estimates for SI remained above 1 but were not statistically significant.

**Conclusion:**

Heavy physical workload and low decision authority were separately associated with DP. The combination of heavy PWL and low decision authority was often associated with higher risks of DP than would be expected from adding the effects of the single exposures. Increasing decision authority among workers with heavy PWL could help reduce the risk of DP.

**Supplementary Information:**

The online version contains supplementary material available at 10.1007/s00420-023-01983-8.

## Introduction

Globally, the proportion of people over the age of 60 is growing. As in other high-income countries, Sweden’s response to this trend is to adopt strategies aimed at retaining more workers in the labour market, such as prolonging retirement age. However, the high rate of health-related exit from the labour market for example through a disability pension (DP), which is especially high among blue collar workers, risks undermining these strategies (Kadefors et al. 2018).

In Sweden, mental disorders are the main diagnosis category for DP among men and women and accounted for around 50% of new DP cases in 2021. Other common categories include diseases of the nervous system, circulatory system, and musculoskeletal disorders. In 2021, musculoskeletal disorders made up 12% of cases among women and 7% among men (Försäkringskassan 2022).

Strenuous working conditions such as heavy physical workload (Ervasti et al. 2019; Falkstedt et al. 2021; Kjellberg et al. 2016; Halonen et al. 2020) and poor psychosocial factors (Christensen et al. 2008; Knardahl et al. 2017; Sundstrup et al. 2018; Leineweber et al. 2019) have been associated with DP. The fact that physical and psychosocial workplace factors often co-occur has been recognised in the literature (Punnett and Wegman 2004). Strong negative correlations between psychosocial factors (such as decision latitude) and physical load have been found among blue-collar workers and low-level office workers (MacDonald et al. 2001). One explanation for this could be the organisation of the work process among different working groups. High paced work, which is typically found among workers with physically demanding jobs, can be driven by production outcomes or wages. An example of this is piece-rate payment rather than hourly wages, which can introduce time pressures. This high paced work could decrease recovery time between tasks or restrict task variation thus limiting opportunities to alternate muscle groups and avoid over exertion (Punnett and Wegman 2004). Examining the combined effects of heavy physical workload and low decision authority on DP could provide increased insights into how to prevent DP (specifically because of poor musculoskeletal health) among workers.

 Combined exposure to heavy physical work and poor psychosocial factors has been  associated with an increased risk of poorer musculoskeletal health (Devereux et al. 2002; Thorbjörnsson et al. 2000; Widanarko et al. 2014, 2015). However, only a few studies have examined the combined effect of heavy physical workload and low job control on health-related exit from the labour market (Gustafsson et al. 2020; Helgesson et al. 2020; Sundstrup and Andersen 2021; Andersen et al. 2020). A Swedish study found that the risks of DP among care assistants and workers in all other occupations (not nurses or care assistants) with combined exposure to heavy physical work and low job control exceeded the sum of the effects of each exposure separately, but not for nurses (Gustafsson et al. 2020). A Danish study found an association between heavy physical workload and DP among female eldercare workers but not between low Influence at work and DP (Andersen et al. 2020). Two studies have investigated the effects of physical workload and low job control on sickness absence (Helgesson et al. 2020; Sundstrup and Andersen 2021). Neither study found statistically significant evidence for an interaction between strenuous physical work and lower job control on sickness absence. In fact, the Danish study found that the risk of sick leave appeared greater among workers with heavy physical workload and good influence at work (Sundstrup and Andersen 2021).

It should be noted that the level and distribution of exposures to workplace factors can vary by gender, thus studies on combined exposure to physical and psychosocial exposures would benefit from gender-stratified analyses (Sabbath et al. 2013). Evidence on the gender-specific combined effects of physical workload and decision authority on DP is, however, lacking.

In this study, we hypothesised that low decision authority exacerbates the effect of heavy physical workload on the risk of DP (all-cause or musculoskeletal). Therefore, we investigated the separate and combined effects of heavy physical workload and low decision authority on DP, separately for men and women.

## Method

### Study population

This study uses a sample of workers from the register-based Swedish Work, Illness, and labour-market Participation (SWIP) cohort that includes all individuals 16–64 years of age who were registered as living in Sweden in 2005, approximately 5.4 million people. The cohort is formed through the linkage of data from several registers, which is made possible through the unique personal identity numbers assigned to registered persons living in Sweden. Statistics Sweden (SCB) collated and deidentified the data to protect confidentiality. Details of the SWIP cohort have previously been published (Falkstedt et al. 2021). The registers used in this study are the Swedish total population register, the Longitudinal Integrated Database for Health Insurance and Labour Market Studies register (LISA), the Micro Data for Analysis of the Social Insurance System (MIDAS) and the Swedish National inpatient register. The data obtained from each register are described below.

### Participants and study design

Workers born between 1946 and 1965 (44–63 years old at the 2009 baseline) were selected for this study (2,378,039). This age group was chosen to try to capture those most at risk of a DP and those still eligible (below 65-year-old) to claim a DP during the follow-up period, 01/01/2010 to 31/12/2019. A worker was defined as an individual with a Swedish Standard Classification of Occupation (SSYK) 96 code. The SSYK codes are used to classify occupations (SCB, 2001) and were obtained from the LISA register for all study participants.

**Fig. 1:**
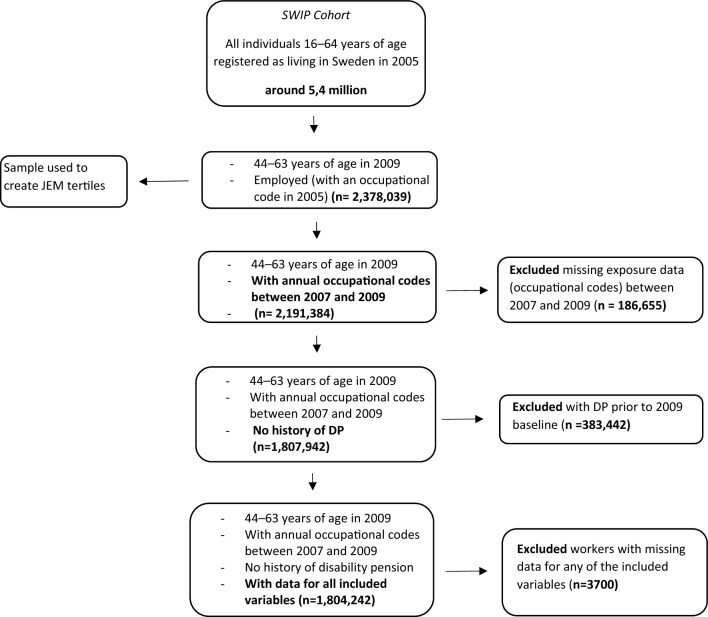
Sample selection

Workers were excluded from the sample if they were missing an SSYK code for any of the years between 2007 and 2009 (*n* = 186,655), had a DP during or prior to the 2009 baseline (*n* = 383,442, which consisted of more women than men), or missing data for any of the included variables (*n* = 3700). The final sample included 1,804,242 workers (932,467 men and 871,775 women), Fig. 1.

### Physical workload (exposure)

Exposure to physical workload was estimated using a Swedish Job Exposure Matrix (JEM). The JEM provides a gender-specific aggregated measure of exposure to overall physical workload (Index score) for 355 occupations. The JEM was constructed using the responses to eight questions on different ergonomic exposures (heavy lifting (≥ 15 kg), physically strenuous work, fast breathing due to physical workload, forward bent position, twisted position, working with hands above shoulder level, repetitive work and frequent bending and twisting) included in the Swedish Work Environment Surveys (1997–2013) (Badarin et al. 2021). An index score (overall physical workload) was created by summing the scores for each of the eight physical workload exposures and calculating a mean value. In this study, the mean JEM values for the index score (overall PWL) were assigned to the SSYK codes of all participants for the years 2007 to 2009. The mean values for the participants for each year were then averaged across the three years (2007 to 2009) to identify a mean level of exposure. This approach was chosen to try to account for any changes in exposure before the start of follow-up. Last, gender-specific variables were created using tertile cut-offs to estimate high, medium, and low exposure to physical workload.

### Decision authority at work (exposure)

Exposure to decision authority at work was estimated using a Swedish JEM for psychosocial workload. The JEM was developed on the same material and with the same procedure as the physical JEM and has been previously described (Almroth et al. 2021). The JEM provides a gender-specific mean index score for decision authority based on responses to four questions on perceived control over when tasks are conducted, work pace, work breaks and work structure. The JEM scores for decision authority are linked to occupations using the SSYK 96 coding system. The index scores for decision authority were fixed to SSYK codes (from LISA) for each participant in this study for the years 2007 to 2009. The mean values for each year were then averaged across the three years (2007 to 2009) to identify a mean level of exposure for decision authority. Gender-specific variables were created using tertile cut-offs to estimate low, medium, and high exposure. It should be noted that decision authority and skill discretion (the breadth of skills a worker can use in his/her job) are linked under the umbrella of ‘decision latitude’ in Karasek’s and Theorell’s classic ‘Job Demand-Control-Model’ (Karasek, 1979, 1997). However, a JEM on skill discretion was not available for use in this study.

### Combined exposure to physical workload and decision authority at work

To investigate the combined effects of exposure to overall physical work and decision authority on the risk of DP, the tertiles for physical workload were combined with the tertiles for decision authority thus creating a new exposure variable with nine-levels as shown in Table 1.

**Table 1:** Nine-level exposure variable for combined exposure to decision authority and heavy physical workload

i	High decision authority and low physical workload (reference category)
ii	High decision authority and medium physical workload
iii	High decision authority and high physical workload
iv	Medium decision authority and low physical workload
v	Medium decision authority and medium physical workload
vi	Medium decision authority and high physical workload
vii	Low decision authority and low physical workload
viii	Low decision authority and medium physical workload
ix	Low decision authority and high physical workload

### Disability pension (DP) (outcome)

All persons aged 30 to 64 years old who, due to illness, injury, or disability, have a medically certified permanent reduction in work ability, of at least 25%, are eligible to obtain a DP (Forsakringskassan 2021). Workers with a DP prior to the 2009 baseline were excluded. In 2008, Sweden introduced more stringent eligibility requirements for DP, subsequently the number of granted applications reduced significantly (Kadefors et al. 2018). Therefore, in this study, DP cases were investigated after the 2008 changes in eligibility requirements (between 2010 to 2019). DP can be granted in full or partially (three-quarter, one-half or one-quarter) depending on one’s work ability. Any first time, full or partial DP were included as a case. Information on DP were obtained from the MiDAS register and two outcomes were explored: all-cause DP (any ICD 10 code) and DP due to a musculoskeletal diagnosis (ICD 10 codes M00-M99).

### Covariates

Potential confounders were identified through existing literature. With restricted information on lifestyle factors in the register cohorts, the chosen covariates provide a proxy indication for lifestyle factors and jointly capture important variations in risk of DP among workers with heavy physical work and/or low job control. The following variables were taken from the LISA register for the baseline year 2009. Educational attainment, which was divided into four groups: (i) primary and lower secondary school or less (≤ 9 years); (ii) secondary (10–11 years); (iii) upper-secondary (12 years); (iv) tertiary (≥ 13 years). Civil status was categorised as either married, unmarried, divorced, or widowed. Country of birth was dichotomised into born in or out of Sweden. Unemployment five years before the start of follow-up was divided into three groups: (i) 0 days (ii) 1–365 days and (iii) > 365 days. Last, the in-patient register provided data on history of hospitalisation for a psychiatric illness five years prior to start of follow-up, which was identified using ICD 10 codes F00 to F99.

### Statistical analysis

First, we examined the sex-specific distribution of the covariates across the exposure’s physical workload and decision authority. Second, associations between the covariates and all-cause and musculoskeletal DP were assessed separately for men and women using Cox proportional-hazards regression, which produces hazard ratios (HR) with 95% confidence intervals (95% CI). Third, we investigated the independent effects of exposure to medium or heavy vs. low physical workload, or low or medium vs. high decision authority on the risk of all-cause and musculoskeletal DP using HR and 95% CI. Fourth, we investigated the combined effect of overall physical work and decision authority on the risk of DP using the nine-level categorical variables. Crude and adjusted HR with 95%CI were used to examine confounding. For the Cox regression analyses person-time was calculated from 1^st^ January 2010 until either emigration, turning 65 years old, death, DP or the end of follow-up on 31st December 2019. Model 1 shows the crude results (adjusted for age) and Model 2 is adjusted for all selected confounders. Finally, interaction effects between heavy physical workload and low decision authority were explored using the synergy index (SI) first presented by Rothman (1986).

In this study, the SI measures how much the effect of combined exposure to heavy physical workload and low decision authority on DP exceeds the sum of the effects of each exposure separately when those unexposed to both exposures are used as reference category (Andersson et al. 2005; VanderWeele and Knol 2014). The SI is defined as:

$$SI= \frac{{RR}_{11}-1}{\left({RR}_{10}-1\right)+({RR}_{01}-1)}$$

In this study, the SI was calculated using the relative risks (RR) from the following exposure categories:

$$SI = \frac{{RR_{{{\text{low DA }}\& {\text{ high PWL }}}} - 1}}{{\left( {RR_{{{\text{low DA }}\& {\text{ low PWL}}}} - 1} \right) + \left( {RR_{{{\text{high DA }}\& {\text{ high PWL}}}} - 1} \right)}}$$

The estimates for high physical workload and low decision authority were used to calculate the SI because these were more contrasting exposure categories and could result in less misclassification than using the medium exposure category. If the SI is greater than one, a positive synergistic interaction is implied. The 95% CI for the SI were calculated according to Andersson et al. (2005) (Andersson et al. 2005). All statistical analyses were conducted using SAS version 9.4.

## Results

During the follow-up, 42,019 cases of all cause DP were identified (19,956 among men and 23,063 among women), of which 8342 cases (3169 among men and 5173 among women) were because of a musculoskeletal diagnosis. The top 10 most commonly occurring diagnoses for all-cause DP or musculoskeletal DP among men and women in the included sample are shown in supplementary material 1. The mean follow-up time was 8.98 years for men and 9.07 years for women.

For both genders, a higher proportion of younger workers, workers born outside of Sweden, with primary or secondary education, unmarried or with a history of unemployment were among those with a high level of heavy physical work compared to a low level of physical workload (Table 2). Among women, the proportion of workers with a history of psychiatric illness was similar among all exposure groups for physical work, but among men the proportion was slightly higher among those with medium or high physical work compared to those with low physical work.

**Table 2:** Distribution of baseline covariates according to physical workload and decision authority for men and women

	Physical workload					
	Men (*n* = 932,467)			Women (*n* = 871,775)		
	Low (*n* = 321,613)	Medium (*n* = 305,743)	Low (*n* = 321,613)	Medium (*n* = 305,743)	Low (*n* = 321,613)	Medium (*n* = 305,743)
	*n* (%)	*n* (%)	*n* (%)	*n* (%)	*n* (%)	*n* (%)
Age						
44–48	88,614 (28)	82,996 (27)	90,248 (30)	85,509 (28)	81,813 (28)	79,029 (29)
49–53	76,842 (24)	76,696 (25)	78,431 (26)	74,345 (24)	75,302 (26)	70,246 (26)
54–58	75,626 (24)	73,361 (24)	70,108 (23)	72,830 (24)	70,343 (24)	62,169 (23)
59–63	80,531 (25)	72,690 (24)	66,324 (22)	76,082 (25)	66,192 (23)	57,915 (22)
Country of birth						
Sweden	295,751 (92)	269,315 (88)	258,871 (85)	280,091 (91)	263,982 (90)	217,193 (81)
Other	25,862 (8)	36,428 (12)	46,240 (15)	28,675 (9)	29,668 (10)	52,166 (19)
Educational level^a^						
Primary	18,925 (6)	58,809 (19)	99,009 (33)	15,532 (5)	24,764 (8)	60,057 (22)
Secondary	55,041 (17)	113,814 (37)	151,187 (50)	59,307 (19)	98,811 (34)	140,024 (52)
Upper-secondary	47,201 (15)	48,261 (16)	32,947 (11)	38,826 (13)	38,366 (13)	40,122 (15)
Tertiary	200,446 (62)	84,859 (28)	21,968 (7)	195,101 (63)	131,709 (45)	29,156 (11)
Civil status						
Married	212,434 (66)	170,131 (56)	152,111 (50)	186,042 (60)	172,372 (59)	143,979 (54)
Unmarried	62,868 (20)	81,316 (27)	99,786 (33)	59,311 (19)	61,302 (21)	61,377 (23)
Divorced	43,552 (14)	51,498 (17)	50,545 (17)	56,871 (18)	53,136 (18)	55,736 (21)
Widowed	2759 (1)	2798 (1)	2669 (1)	6542 (2)	6840 (2)	8267 (3)
Hospitalisation for a psychiatric illness*						
No	318,790 (99)	301,153 (99)	299,125 (98)	306,455 (99)	290,828 (99)	265,357 (98)
Yes	2823 (1)	4590 (2)	5986 (2)	2311 (1)	2822 (1)	4002 (2)
Unemployment*						
0	289,611 (90)	250,608 (82)	231,222 (76)	272,630 (88)	245,885 (84)	210,845 (78)
1–365	22,848 (7)	38,429 (13)	50,898 (17)	27,500 (9)	35,371 (12)	42,406 (16)
> 365	9154 (3)	16,706 (6)	22,991 (8)	8636 (3)	12,394 (4)	16,108 (6)

	Decision authority					
	Men (*n* = 932,467)			Women (*n* = 871,775)		
	Low (*n* = 310,425)	Medium (*n* = 301,271)	High (*n* = 320,771)	Low (*n* = 286,257)	Medium (*n* = 282,823)	High (*n* = 302,695)
Age						
44–48	88,403 (29)	85,396 (28)	88,059 (28)	80,134 (28)	77,886 (28)	88,331 (29)
49–53	79,667 (26)	74,898 (25)	77,404 (24)	73,112 (26)	72,744 (26)	74,037 (25)
54–58	73,024 (24)	70,717 (24)	75,354 (24)	68,813 (24)	67,619 (24)	68,910 (23)
59–63	69,331 (22)	70,260 (23)	79,954 (25)	64,198 (22)	64,574 (23)	71,417 (24)
Country of birth						
Sweden	257,694 (83)	267,390 (89)	298,853 (93)	242,833 (85)	243,494 (86)	274,939 (91)
Other	52,731 (17)	33,881 (11)	21,918 (7)	43,424 (15)	39,329 (14)	27,756(9)
Educational level^a^						
Primary	78,531 (25)	61,203 (20)	37,009 (12)	35,418 (12)	40,876 (15)	24,059 (8)
Secondary	117,513 (38)	128,546 (43)	73,983 (23)	119,135 (42)	100,524 (36)	78,483 (26)
Upper-secondary	36,988 (12)	43,360 (14)	48,061 (15)	34,433 (12)	35,852 (13)	47,029 (16)
Tertiary	77,393 (25)	68,162 (23)	161,718 (50)	97,271 (34)	105,571 (37)	153,124 (51)
Civil status						
Married	161,900 (52)	164,150 (55)	208,626 (65)	162,188 (57)	163,043 (58)	177,162 (59)
Unmarried	91,616 (30)	85,637 (28)	66,717 (21)	59,911 (21)	59,180 (21)	62,899 (21)
Divorced	54,199 (18)	48,763(16)	42,633 (13)	56,423 (20)	53,325 (19)	55,995 (19)
Widowed	2710 (1)	2721 (1)	2795 (1)	7735 (2)	7275 (3)	6639 (2)
Hospitalisation for a psychiatric illness*						
No	304,890 (98)	296,471 (98)	317,707 (99)	282,554 (99)	279,718 (99)	300,368 (99)
Yes	5535 (2)	4800 (2)	3064 (1)	3703 (1)	3105 (1)	2327 (1)
Unemployment*						
0	243,096 (78)	241,995 (80)	286,350 (89)	238,963 (84)	230,355 (81)	260,042 (86)
1–365	47,102 (15)	40,739 (14)	24,334 (8)	35,981 (13)	38,448 (14)	30,848 (10)
> 365	20,227 (7)	18,537 (6)	10,087 (3)	11,313 (4)	14,020 (5)	11,805 (4)

^**a**^Primary =  ≤ 9 years; secondary = 10–11 years; upper-secondary = 12 years; tertiary =  ≥ 13 years

*5 years prior to start of follow-up (2010)

The distribution of the age groups among the different levels of decision authority were similar for both sexes (Table 2). Among workers with lower decision authority (low and medium), the proportions of workers born outside of Sweden or with primary and secondary level education were higher than among those with high decision authority. Being unmarried was more prevalent among workers with low decision authority for men, but not women. A higher proportion of workers with low (and medium for women) decision authority had a history of unemployment compared to those with high decision authority.

Being older, born outside of Sweden, having lower educational attainment, a hospitalisation for a psychiatric illness, being divorced, or previous unemployment were individually associated with increased risks of DP and musculoskeletal DP for both sexes (Table 3). Apart from for a hospitalisation for a psychiatric illness, the relative risks were higher for musculoskeletal DP than all-cause DP.

**Table 3:** The association between the included confounders and all cause disability pension and musculoskeletal disability pension

	All cause disability pension		Musculoskeletal disability pension	
	Men (*n* = 932,467)	Women (*n* = 871,775)	Men (*n* = 932,467)	Women (*n* = 871,775)
	HR (95% CI)	HR (95% CI)	HR (95% CI)	HR (95% CI)
Age				
44–48	1	1	1	1
49–53	1.63(1.57–1.70)	1.36(1.32–1.41)	1.64(1.48–1.82)	1.45(1.34–1.56)
54–58	2.31(2.22–2.40)	1.69(1.63–1.75)	2.49(2.25–2.75)	1.85(1.72–1.99)
59–63	2.65(2.52–2.79)	1.75(1.67–1.83)	3.18(2.81–3.58)	1.86(1.69–2.05)
Country of birth				
Sweden	1	1	1	1
Other	1.77(1.71–1.83)	1.71(1.65–1.76)	2.25(2.07–2.44)	2.05(1.92–2.19)
Educational level ^a^				
Tertiary	1	1	1	1
Upper-secondary	1.58 (1.50–1.66)	1.48 (1.42–1.55)	2.26 (1.96–2.61)	2.14 (1.94–2.35)
Secondary	2.03 (1.95–2.12)	1.72 (1.67–1.78)	3.50 (3.13–3.91)	2.74 (2–55-2.95)
Primary	2.42 (2.33–2.53)	2.39 (2.30–2.49)	4.55 (4.05–5.11)	4.29 (3.94–4.67)
Civil status				
Married	1	1	1	1
Unmarried	1.52(1.47–1.57)	1.17(1.14–1.21)	1.09(1.00–1.19)	0.86(0.79–0.92)
Divorced	1.67(1.61–1.73)	1.51(1.46–1.56)	1.45(1.33–1.59)	1.31(1.22–1.40)
Widowed	1.61(1.40–1.85)	1.24(1.14–1.36)	1.51(1.08–2.10)	1.13(0.94–1.35)
Hospitalisation for a psychiatric illness*				
No	1	1	1	1
Yes	8.09(7.71–8.49)	8.44(8.03–8.87)	3.43(2.90–4.06	2.57(2.17–3.06)
Unemployment*				
0	1	1	1	1
1–365	2.42(2.34–2.51)	2.25(2.18–2.33)	2.90(2.68–3.15)	2.48(2.32–2.65)
> 365	3.18(3.04–3.32)	3.02(2.89–3.16)	3.28(2.94–3.66)	3.02(2.75–3.31)

^a^Primary =  ≤ 9 years; secondary = 10–11 years; upper–secondary = 12 years; tertiary =  ≥ 13 years

*5 years prior to start of follow-up (2010)

All models adjusted for age

For both sexes, compared to workers in the lowest tertile, medium or high exposure to overall physical workload was associated with an increased risk of all-cause DP and musculoskeletal DP (Table 4). The highest exposure was associated with the greatest increased risks of DP indicating a dose response-like relationship between heavy physical work and DP. These associations remained after adjusting for age, education, civil status, country of birth and hospitalization for a psychiatric illness. Compared to workers in the highest tertile, the risks of DP among workers with low or medium decision authority were similar (Table 4). A clear dose response-like relationship was not found between decision authority and DP. Overall, the highest increased risks of DP were found for associations between high exposure to heavy physical work or low or medium decision authority and musculoskeletal DP (Table 4).

**Table 4:** Hazard ratios and 95% confidence intervals for the association between all cause and musculoskeletal disability pension and separate exposure to physical workload and decision authority among men and women *n* = 1,804,242

	All cause DP			MSD DP		
	n of cases	Model 1	Model 2	n of cases	Model 1	Model 2
Physical workload						
Men						
Low	3587/321613	1	1	321/321613	1	1
Med	6787/305743	1.98(1.90–2.06)	1.49(1.43–1.56)	994/305743	3.23(2.85–3.66)	2.20 (1.93–2.51)
High	9582/305111	2.82(2.71–2.93)	1.77(1.69–1.85)	1854/305111	6.09(5.41–6.85)	3.29 (2.89–3.76)
Women						
Low	4909/308766	1	1	711/308766	1	1
Med	7194/293650	1.52(1.47–1.58)	1.36(1.31–1.41)	1478/293650	2.16(1.97–2.36)	1.82(1.67–2.00)
High	10,960/269359	2.55(2.47–2.64)	1.83(1.76–1.90)	2984/269359	4.78(4.40–5.19)	2.98(2.72–3.25)
Decision authority						
Men						
Low	8500/310425	2.14(2.06–2.22)	1.49(1.44–1.55)	1439/310425	3.00(2.71–3.33)	1.86 (1.68–2.07)
Med	7355/301271	1.91(1.84–1.99)	1.41(1.36–1.47)	1237/301271	2.67(2.40–2.96)	1.77 (1.59–1.97)
High	4101/320771	1	1	493/320771	1	1
Women						
Low	9046/286257	1.80(1.74–1.86)	1.52(1.47–1.57)	2170/286257	2.57(2.37–2.77)	2.02(1.86–2.19)
Med	8744/282823	1.76(1.70–1.83)	1.50(1.45–1.55)	2120/282823	2.54(2.35–2.75)	2.01(1.85–2.17)
High	5273/302695	1	1	883/302695	1	1

Model 1-adjusted for age

Model 2–adjusted for age, education, civil status, country of birth, hospitalisation for a psychiatric illness and unemployment

In unadjusted models, men and women with combined exposure to overall heavy physical workload and low or medium decision authority had greater risks of all-cause DP or musculoskeletal DP than when adding the effects of the single exposures (Table 5). The results for the SI were larger than one for all-cause DP (men: SI 1.35 95%CI 1.18–1.55, and women: SI 1.19 95%CI 1.05–1.35,) and musculoskeletal disorder DP (men: SI 1.35 95%CI 1.08–1.69, and women: SI: 1.13 95%CI 0.85–1.49). After adjustment, the estimates for SI remained above 1 but were not statistically significant.

**Table 5:** Hazard ratios (HR) and 95% confidence intervals for the association between DP (all cause and musculoskeletal) and combined exposure to overall physical workload (PWL) and decision authority (DA) among men and women *n* = 1,804,242

	Decision authority					
	Model 1			Model 2		
	High	Med	Low	High	Med	Low
All Cause DP						
Men Overall PWL						
Low	1	1.39(1.28–1.50)	1.60(1.45–1.75)*	1	1.26(1.16–1.36)	1.53(1.39–1.68)*
Med	1.98(1.85–2.12)	2.16(2.04–2.28)	2.54(2.40–2.69)	1.56(1.45–1.67)	1.60(1.51–1.70)	1.79(1.68–1.89)
High	2.05(1.88–2.24)*	3.52(3.34–3.71)	3.23(3.07–3.39)*	1.48(1.35–1.62)*	2.11(1.99–2.24)	1.96(1.86–2.08)*
SI			**1.35 (1.18–1.55)**			**1.04 (0.87–1.25)**
Women Overall PWL						
Low	1	1.17(1.09–1.27)	1.21(1.11–1.31)*	1	1.22(1.13–1.32)	1.34(1.23–1.46)*
Med	1.51(1.42–1.61)	1.72(1.64–1.79)	1.36(1.27–1.44)	1.32(1.24–1.41)	1.53(1.46–1.60)	1.36(1.28–1.45)
High	2.03(1.83–2.25)*	3.42(3.26–3.58)	2.46(2.37–2.57)*	1.48(1.33–1.64)*	2.24(2.13–2.36)	1.87(1.79–1.96)*
SI			**1.19 (1.05–1.35)**			**1.12 (0.88–1.42)**
MSD DP						
Men Overall PWL						
Low	1	1.88(1.47–2.42)	1.83(1.34–2.50)*	1	1.66(1.30–2.14)	1.88(1.38–2.57)*
Med	3.63(2.97–4.46)	3.87 (3.24–4.63)	4.62(3.87–5.51)	2.58(2.09–3.17)	2.60(2.16–3.12)	2.96(2.47–3.55)
High	5.08(4.03–6.39)*	8.61(7.31–10.15)	7.61(6.48–8.94)*	3.25(2.56–4.12)*	4.46(3.74–5.31)	3.98(3.36–4.73)*
SI			**1.35 (1.08–1.69)**			**1.03 (0.46–2.31)**
Women Overall PWL						
Low	1	1.05(0.86–1.29)	1.00(0.79–1.27)*	1	1.14(0.93–1.40)	1.26(0.99–1.60)*
Med	1.77(1.52–2.06)	2.48(2.23–2.76)	1.73(1.49–2.01)	1.45(1.24–1.69)	2.11(1.90–2.35)	1.79(1.54–2.08)
High	3.85(3.15–4.72)*	6.73(6.05–7.48)	4.21(3.82–4.64)*	2.55(2.08–3.13)*	3.85(3.43–4.31)	2.88(2.60–3.19)*
SI			**1.13 (0.85–1.49)**			**1.04 (0.83–1.59)**

Model 1-adjusted for age

Model 2–adjusted for age, education, civil status, country of birth,hospitalisation for a psychiatric illness and unemployment

SI = Synergy Index for high exposure heavy PWL and low decision authority, results for the SI are shown in bold

HR with *were used to calculate the SI

Baseline 2009

## Discussion

### Summary

This study investigates the separate and combined effects of heavy physical workload and low decision authority on DP among male and female workers in the Swedish population. Among both sexes, the association between heavy physical workload and DP followed a dose–response like pattern, but this pattern was not as clear for decision authority. The strongest associations were found for musculoskeletal DP. Workers with combined exposure to heavy physical workload and low (or medium) decision authority often had higher risks of DP (all-cause and musculoskeletal) than when adding the effects of the single exposures. The results for the SI were higher than 1 indicating a more than additive relationship between heavy physical workload and low decision authority. However, after adjustment, the estimates for the SI were not statistically significant.

### Comparison to other studies

In this study, the observed increased risks of DP associated with exposure to heavy physical workload and lower levels of decision authority are in agreement with previous literature (Falkstedt et al. 2021; Kjellberg et al. 2016; Ervasti et al. 2019; Sundstrup et al. 2018; Knardahl et al. 2017; Christensen et al. 2008).

We also found that the risks of DP among male and female workers with combined exposure to heavy physical workload and low or medium decision authority often appeared higher than when adding the effects of the single exposures. The results for the SI were larger than one, which suggests that the relationship between heavy physical workload and low decision authority is more than additive. However, after adjustment, the results for the SI were not statistically significant.

The combined effect of heavy physical workload and low job control on health-related exit from the labour market has only been investigated in a small number of studies. A Danish study on around 4000 healthy female healthcare workers found an association between heavy physical workload and DP but did not find an association between low influence at work and DP (Andersen et al. 2020). Our findings are somewhat in line with the findings of an earlier Swedish study that investigated the combination of heavy physical work and low job control on all-cause DP among three occupational groups: nurses and care assistants (92% women), and workers in any other occupations (54% men) (Gustafsson et al. 2020). The study found that the risks of DP among healthcare assistants and workers in the other occupations with combined exposure to heavy physical workload and low job control often appeared higher than when adding the effects of the single exposures, but this was not found among nurses. The results for the SI for the combination of heavy physical work and low decision authority were larger than one for care assistants and workers in any other occupation. Furthermore, unlike the results for the SI in this present study, after adjustment the results for the SI were statistically significant for those in the other occupations group.

Several methodological differences in this current study could partly explain why our findings are discordant with the results in the existing literature. First, we conducted separate analysis for men and women. Second, our sample included the general Swedish working population unlike many of the existing studies that focussed on healthcare workers. Third, we investigated both all-cause and musculoskeletal disorder DP. Last, we used a trichotomised rather than a dichotomised exposure classification, which offers more contrasting groups that are less likely to be misclassified.

The indication of a more than additive relationship between heavy physical workload and low decision authority on DP found in this study is in line with existing literature showing an increased risk of poorer musculoskeletal health among workers with combined exposure to heavy physical work and poor psychosocial factors (Devereux et al. 2002; Thorbjörnsson et al. 2000; Widanarko et al. 2014, 2015). However, existing studies on the combinations of heavy physical workload and low job control on sick leave have found varying results. A Swedish study found that care assistants with heavy physical workload and low job control appeared to have higher risks of sickness absence than when adding the effects of the single exposures, but this was not found among nurses (Helgesson et al. 2020). Furthermore, a Danish study found that, compared to workers with low physical workload and good influence at work, the risk of long term sick leave was highest among workers with heavy physical workload and good influence at work (Sundstrup and Andersen 2021). The outcome used in this study (DP) differs to those used in many of the existing studies (musculoskeletal pain or sick leave), which means the results are not directly comparable. However, overall, the findings of this current study are in agreement with the existing literature that underscores the need to focus on combined rather than single workplace risk factors to ensure the health of workers.

### Strengths and weaknesses

A strength of this study is the large population-based sample, which increases the generalisability of our findings within regions around Sweden and to countries with similar labour markets and social welfare systems. Moreover, register-based cohort studies do not suffer from participation or attrition bias that can be found in cohort studies using survey data. Further strengths include the studies prospective design and long follow-up period. It should also be noted that the outcome, DP, was taken from an administrative source and differential misclassification of the outcome is unlikely. Our results also build upon earlier literature to show sex-specific risks associated with combined exposure to heavy physical workload and low decision authority and DP.

Using the JEMs to measure the workplace exposures is another strength of this study. The application of the JEMs helps to reduce common method bias that can occur from the use of self-report data, as the data used to create the JEMs are taken from a different sample than the one under investigation. However, it should be noted that the JEMs are constructed on self-reported data on exposure to physical workload, which is typically perceived as less accurate than more objective methods e.g., accelerometery (Wells et al. 1997). Using an index value to measure physical workload enabled us to estimate the effects of combined exposure to low job control and overall heavy physical workload. Furthermore, the use of tertiles for exposure to physical workload and job control provide more contrasting exposure groups that are less likely to be misclassified compared to using dichotomised exposures.

It is important to note some limitations of this study. The JEMs provide a measure of workplace exposures at a group level based on occupation. Thus, differences in exposure to physical workload or job control between workers within occupations could not be captured in this study. Though we were able to account for a range of potential confounding variables that could obscure the relationship between physical workload, low job control and DP, potential residual or unmeasured confounding should be acknowledged, as information on lifestyle factors e.g., BMI, smoking or leisure time physical activity are not available in this register-based cohort. However, education could be seen as a crude proxy for lifestyle factors, as such factors can differ between socioeconomic groups in Sweden (Mäki et al. 2014). Furthermore, we were unable to account for changes in exposure after the start of follow-up, or lifetime exposure to physical workload factors before the baseline exposure. However, our mean measurement over a three-year period accounted for some fluctuation in exposure prior to the follow-up period.

### Interpretation of results

Our finding that heavy physical workload and lower levels of decision authority were separately associated with an increased risk of DP underscores the need to minimise these workplace hazards to try to protect workers health and prevent DP.

In this study male and female workers with combined exposure to heavy physical work and low decision authority often had higher risks of DP than would be expected from adding each exposure (the SI were often higher than 1). However, for both sexes, after adjusting for age, education, civil status, country of birth, hospitalisation for a psychiatric illness and unemployment the estimates for the SI were no longer statistically significant indicating that this increased additive effect was explained by the included covariates. Existing literature has suggested several mechanisms that could explain the pathways between exposure to heavy physical workload and low job control on musculoskeletal health (Punnett and Wegman 2004, Theorell 2005). Poor psychosocial workplace factors, such as low job control, could act as specific stressors that cause physiological strain on the musculoskeletal system through pathways such as hormonal changes or muscle tension (Theorell 2005). Thus, in combination with exposure to heavy physical workload the risks of poorer musculoskeletal health could be greater than among workers exposed to only one of the exposures. Another explanation is that low job control could aggravate the effect of heavy physical workload on DP as workers cannot adapt their workplace design, working routine or tasks to avoid injuries from stressors such as repetitive or hazardous work posture, or over exertion (Punnett and Wegman 2004). Overtime, workers with heavy physical workload and lower job control could experience a higher accumulation of exposure compared to workers with heavy physical workload and higher job control.

The finding that workers with combined exposure to heavy physical workload and lower levels of decision authority (medium or low) often had higher risks of DP (all-cause and musculoskeletal) than would be expected from adding the effects of the single exposures is in agreement with the hypothesis that lower decision authority aggravates the effect of heavy physical workload on the risk of DP. Increasing job control among workers with heavy physical workload could reduce the risk of DP. For example, allowing workers more autonomy over when to take breaks could help with recovery from biomechanical strain or introducing task variation could help to alternate muscle groups and prevent over exertion. This interpretation, however, is slightly hampered by our finding that the risk of DP among workers with combined exposure to heavy physical workload and low decision were lower than among those with combined exposure to heavy physical workload and medium decision authority. This was a somewhat unexpected result. An examination of the occupations among the different exposure categories showed that domestic workers (helpers and cleaners) made up the largest proportion of female workers with heavy physical workload and medium decision authority (supplementary material 1). Whereas nursing assistants made up a large proportion of female workers with heavy physical workload and low decision authority. One explanation for the lower risks of DP among workers with heavy physical workload and low decision authority is unmeasured confounding by different aspects of the work environment, such as support from colleagues or access to ergonomic equipment. For example, nursing assistants could have more opportunity to work with manual handling equipment or in cooperation with colleagues than cleaners. That said, the same risk pattern was observed among men in the comparable groups, but we found a larger variety of occupations among the exposure groups than among the women (supplementary material 2).

The crude results showed that combined exposure to heavy physical workload and low decision authority were associated with a larger increased risk of musculoskeletal DP than all-cause DP. This was an expected result because of the known association between heavy physical work and musculoskeletal disorders and is in line with the findings of previous studies (Falkstedt et al. 2021). However, the synergy index of relative comparisons showed a similar pattern for both outcomes.

## Conclusion

Among both sexes, heavy physical workload or low decision authority were separately associated with an increased risk of DP (all-cause and musculoskeletal). Workers with combined exposure to heavy physical workload and lower levels of decision authority (medium or low) often had higher risks of DP (all-cause and musculoskeletal) than would be expected from adding the effects of the single exposures (the SI were often higher than 1). However, after adjustment, the estimates for the SI were not statistically significant indicating that this increased additive effect was explained by the included covariates.

## Supplementary Information

Below is the link to the electronic supplementary material.

Supplementary file1 (DOCX 20 KB)

## Data Availability

Data may be obtained from a third party and are not publicly available. The data used for this study were obtained from Statistics Sweden (SCB).
