# Supplementary material for: Combined exposure to heavy physical workload and low job control and the risk of disability pension: A cohort study of employed men and women in Sweden
Source: Int Arch Occup Environ Health. 2023 May 29;96(7):973–84. doi: 10.1007/s00420-023-01983-8 (PMC10361844; doi:10.1007/s00420-023-01983-8)
Supplement: Supplementary file 1 — Supplementary file1 (DOCX 20 KB) [file 420_2023_1983_MOESM1_ESM.docx]

**Supplementary material 1. The 10 most commonly occurring** diagnoses **for all-cause and musculoskeletal disability pension among men and women in the included sample of this study (aged 44-66 in the general Swedish working population)**

**.**

| **Supplementary material 1.** | | | |
| --- | --- | --- | --- |
| **All-cause DP** | | **Musculoskeletal DP** | |
| **Men** | **Women** | **Men** | **Women** |
| **I69** Sequelae of cerebrovascular disease | **F43** Reaction to severe stress, and adjustment disorders | **M54** Dorsalgia | **M79** Other and unspecified soft tissue disorders, not elsewhere classified |
| **F43** Reaction to severe stress, and adjustment disorders | **M79** Other and unspecified soft tissue disorders, not elsewhere classified | **M53** Other and unspecified dorsopathies, not elsewhere classified | **M54** Dorsalgia |
| **I63** Cerebral infarction | **F33** Major depressive disorder, recurrent | **M51** Thoracic, thoracolumbar, and lumbosacral intervertebral disc disorders | **M53** Other and unspecified dorsopathies, not elsewhere classified |
| **M54** Dorsalgia | **I69** Sequelae of cerebrovascular disease | **M48** Other spondylopathies | **M05**  Rheumatoid arthritis with rheumatoid factor |
| **F33** Major depressive disorder, recurrent | **F32**  Depressive episode | **M79** Other and unspecified soft tissue disorders, not elsewhere classified | **M19-** Other and unspecified osteoarthritis |
| **F32** Depressive episode | **F41** Other anxiety disorders | **M75** Shoulder lesions | **M51** Thoracic, thoracolumbar, and lumbosacral intervertebral disc disorders |
| **G20** Parkinson's disease | **M54** Dorsalgia | **M50** Cervical disc disorders | **M06** Other rheumatoid arthritis |
| **F31** Bipolar disorder | **F31** Bipolar disorder | **M19** Other and unspecified osteoarthritis | **M50** Cervical disc disorders |
| **F41** Other anxiety disorders | **G35** Multiple sclerosis | **M47** Spondylosis | **M48** Other spondylopathies |
| **M53** Other and unspecified dorsopathies, not elsewhere classified | **M53** Other and unspecified dorsopathies, not elsewhere classified | **M45** Ankylosing spondylitis | **M17** Osteoarthritis of knee |

**Supplementary material 2. The five most commonly occuring jobs in combined exposure categories for heavy physical workload and low decision authority for the included sample of this study (employed men and women aged 44-66 in Sweden)**

TOP 5 JOBS IN TERTILES EXPOSURE GROUPS (Low PWL/ Low DA)

|  | Men | % | Women | % |
| --- | --- | --- | --- | --- |
| 1 | Medical doctors | 27.93 | Primary education teaching professionals | 76.20 |
| 2 | Primary education teaching professionals | 22.68 | Medical doctors | 19.44 |
| 3 | Teaching professionals, academic subjects | 16.72 | Prison guards | 2.21 |
| 4 | Banking associate professionals | 11.83 | Special education teaching professionals | 1.61 |
| 5 | Social workers and related associate professionals | 5.62 | Teaching professionals, academic subjects | 0.42 |

TOP 5 JOBS IN TERTILES EXPOSURE GROUPS (Low PWL/ Med DA)

|  | Men | % | Women | % |
| --- | --- | --- | --- | --- |
| 1 | Physical and engineering science technicians not elsewhere classified | 15.74 | Banking associate professionals | 17.85 |
| 2 | Mechanical engineering technicians | 15.70 | Teaching professionals, academic subjects | 15.83 |
| 3 | Computer assistants | 13.56 | Government social benefits officials | 14.29 |
| 4 | Buyers | 6.16 | Education methods specialists and related professionals | 9.12 |
| 5 | Administrative secretaries and related associate professionals | 5.22 | Special education teaching professionals | 7.87 |

TOP 5 JOBS IN TERTILES EXPOSURE GROUPS (Low PWL/ High DA) (REF Group)

|  | Men | % | Women | % |
| --- | --- | --- | --- | --- |
| 1 | Technical and commercial sales representatives | 11.91 | Numerical clerks | 11.95 |
| 2 | Computer systems designers, analysts and programmers | 8.35 | Administrative secretaries and related associate professionals | 7.99 |
| 3 | Directors and chief executives | 5.78 | Public service administrative professionals | 6.43 |
| 4 | Public service administrative professionals | 4.62 | Social work professionals | 4.39 |
| 5 | Specialist managers not elsewhere classified | 3.60 | Bookkeepers | 3.93 |

TOP 5 JOBS IN TERTILES EXPOSURE GROUPS (Med PWL/ Low DA)

|  | Men | % | Women | % |
| --- | --- | --- | --- | --- |
| 1 | Bus and tram drivers | 10.92 | Nursing associate professionals not elsewhere classified | 21.29 |
| 2 | Car, taxi and van drivers | 7.92 | Dental nurses | 11.79 |
| 3 | Home-based personal care and related workers | 7.40 | Teaching professionals, artistic and practical subjects | 8.88 |
| 4 | Earth-moving- and related plant operators | 4.88 | District nurses | 8.85 |
| 5 | Police officers and detectives | 4.60 | Medical care nurses | 6.15 |

TOP 5 JOBS IN TERTILES EXPOSURE GROUPS (Med PWL/ Med DA)

|  | Men | % | Women | % |
| --- | --- | --- | --- | --- |
| 1 | Agricultural- or industrial-machinery mechanics and fitters | 8.74 | Child-care workers | 21.14 |
| 2 | Shop salespersons, non-food stores | 8.19 | Pre-primary education teaching associate professionals | 20.46 |
| 3 | Other office clerks | 6.12 | Attendants, psychiatric care | 12.86 |
| 4 | Attendants, psychiatric care | 5.35 | Office secretaries | 9.99 |
| 5 | Electrical mechanics fitters and servicers | 5.14 | Shop salespersons, non-food stores | 8.69 |

TOP 5 JOBS IN TERTILES EXPOSURE GROUPS (Med PWL/ High DA)

|  | Men | % | Women | % |
| --- | --- | --- | --- | --- |
| 1 | Building caretakers | 26.29 | Other office clerks | 42.43 |
| 2 | Managers of small enterprises in wholesale and retail trade, hotels and restaurants, transport and kommunications | 16.10 | Physiotherapists and related associate professionals | 5.69 |
| 3 | Civil engineering technicians | 11.30 | Managers of small enterprises in wholesale and retail trade, hotels and restaurants, transport and kommunications | 5.61 |
| 4 | Managers of small enterprises in manufacturing | 5.92 | Finance and sales associate professionals not elsewhere classified | 4.40 |
| 5 | Managers of small enterprises not elsewhere classified | 5.58 | Recreation officers and related associate professionals | 4.17 |

TOP 5 JOBS IN TERTILES EXPOSURE GROUPS (High PWL/ Low DA)

|  | Men | % | Women | % |
| --- | --- | --- | --- | --- |
| 1 | Heavy truck and lorry drivers | 15.96 | Assistant nurses and hospital ward assistants | 40.91 |
| 2 | Machine-tool operators | 10.55 | Home-based personal care and related workers | 23.86 |
| 3 | Stock clerks and storekeepers | 9.42 | Helpers in restaurants | 7.77 |
| 4 | Other machine operators and assemblers | 5.33 | Shop salespersons, food stores | 6.28 |
| 5 | Rail and road construction workers | 4.19 | Manufacturing labourers | 2.06 |

TOP 5 JOBS IN TERTILES EXPOSURE GROUPS (High PWL/ Med DA)

|  | Men | % | Women | % |
| --- | --- | --- | --- | --- |
| 1 | Carpenters and joiners | 17.28 | Helpers and cleaners in offices, hotels and other establishments | 39.64 |
| 2 | Motor vehicle mechanics and fitters | 9.51 | Cooks | 13.78 |
| 3 | Building and related electricians | 7.26 | Other sales and services elementary occupations | 6.81 |
| 4 | Other sales and services elementary occupations | 7.18 | Stock clerks and storekeepers | 5.35 |
| 5 | Plumbers | 6.47 | Health associate professionals not elsewhere classified | 3.72 |

TOP 5 JOBS IN TERTILES EXPOSURE GROUPS (High PWL/ High DA)

|  | Men | % | Women | % |
| --- | --- | --- | --- | --- |
| 1 | Dairy and livestock producers | 26.27 | Hairdressers, barbers, beauticians and related workers | 22.25 |
| 2 | Painters and related workers | 19.14 | Building caretakers | 13.50 |
| 3 | Field crop and vegetable growers | 16.08 | Dairy and livestock producers | 10.36 |
| 4 | Crop and animal producers | 15.20 | Gardeners, parks and grounds | 9.28 |
| 5 | Forestry and related workers | 6.25 | Crop and animal producers | 6.02 |
